# *Helicobacter pylori* Induces Activation of Human Peripheral γδ+ T Lymphocytes

**DOI:** 10.1371/journal.pone.0019324

**Published:** 2011-04-29

**Authors:** Benedetta Romi, Elisabetta Soldaini, Laura Pancotto, Flora Castellino, Giuseppe Del Giudice, Francesca Schiavetti

**Affiliations:** Novartis Vaccines and Diagnostics Research Center, Siena, Italy; University of Hyderabad, India

## Abstract

*Helicobacter pylori* is a Gram-negative bacterium that causes gastric and duodenal diseases in humans. Despite a robust antibody and cellular immune response, *H. pylori* infection persists chronically. To understand if and how *H. pylori* could modulate T cell activation, in the present study we investigated *in vitro* the interaction between *H. pylori* and human T lymphocytes freshly isolated from peripheral blood of *H. pylori*-negative donors. A direct interaction of live, but not killed bacteria with purified CD3+ T lymphocytes was observed by microscopy and confirmed by flow cytometry. Live *H. pylori* activated CD3+ T lymphocytes and predominantly γδ+ T cells bearing the TCR chain Vδ2. Upon interaction with *H. pylori*, these cells up-regulated the activation molecule CD69 and produced cytokines (such as TNFα, IFNγ) and chemokines (such as MIP-1β, RANTES) in a non-antigen-specific manner. This activation required viable *H. pylori* and was not exhibited by other Gram-negative bacteria. The cytotoxin-associated antigen-A (CagA), was at least partially responsible of this activation. Our results suggest that *H. pylori* can directly interact with T cells and modulate the response of γδ+ T cells, thereby favouring an inflammatory environment which can contribute to the chronic persistence of the bacteria and eventually to the gastric pathology.

## Introduction

*Helicobacter pylori* (*H. pylori*) is a spiral shaped Gram-negative bacterium that causes gastric and duodenal disorders. The *H. pylori* infection is typically acquired in early childhood via person-to-person spread, via oral-oral or fecal-oral transmission. The majority of infected individuals remain asymptomatic, and only a 5–15% develops serious complications. Chronic infection with *Helicobacter pylori* is the major known risk factor for duodenal and gastric ulcer diseases and cancer [1], [2], which are frequently associated with the expression of CagA antigen [3], [4], [5].

*Helicobacter pylori* infection induces a strong local immune response with infiltration of the mucosa by neutrophils, macrophages and lymphocytes. Many studies reported that the T cell response to *H. pylori* is prevalently of Th1 type with infiltration of IFN-γ producing T cells in the site of infection [6]. In addition, unconventional T cell populations may also intervene at the mucosal level in response to *H. pylori* stimuli and modulate the outcome of the infection, leading to local inflammation, chronic persistence of lesions and eventually cancer [1]. Some studies have described the involvement of γδ+ T cells in *Helicobacter pylori* gastritis [7], [8], [9]. In particular, one study has reported the infiltration of γδ+ T cells in *H. pylori* infected biopsies that were significantly higher in grade III gastritis while strongly decreased after eradication therapy [10]. Moreover γδ+ T cells appear to have both pro-inflammatory and regulatory functions: they can act as a bridge between innate and adaptive immunity early in the response and down-modulate inflammation once the infection is cleared [7].

In the present study we investigated the interaction of *H. pylori* with human T cell populations, including γδ+ T cells and how this interaction modulated their state of activation and ability to produce cytokines.

## Results

### *H. pylori* directly interacts with T lymphocytes

**Figure 1:**
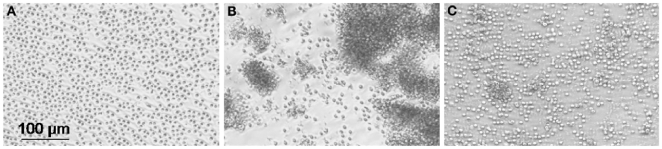
*H. pylori* and T cells co-culture. Viable, but not fixed *H. pylori* cause T cells clustering after 4 h of co-culture. Light microscopy of purified CD3+ cells after 4 h of culture without (A) or with (B) viable *H. pylori* or formaldehyde fixed *H. pylori* (C) (MOI 100).

To investigate whether *H. pylori* physically interacted with human T cells, T lymphocytes were purified from peripheral blood of *H. pylori* negative donors and co-cultured with viable G27 *H. pylori* strain. After 4 h of culture cell clustering was visible microscopically suggesting a direct interaction between T lymphocytes and the live bacteria (Figure 1B). In contrast, formaldehyde fixed *H. pylori* were unable to exert the same effect (Figure 1C). Lymphocyte activation was also evident by cytofluorimetric analysis because of an increase of cellular complexity (side scatter) of T cells cultured with bacteria, as compared to unstimulated control (data not shown).

**Figure 2:**
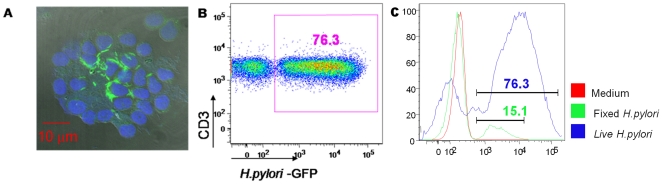
*H. pylori*-GFP and T cells interaction. Interaction of purified human CD3+ T lymphocytes with *H. pylori*-GFP was observed by confocal microscopy (A) after 4 h of co-culture with viable *H. pylori* (MOI 100). T cell nuclei were labeled with DAPI (blue), while green fluorescence belongs to GFP-*H. pylori*. The interaction between T cells and *H. pylori* was observed by FACS analysis (B). The gating was made on live CD3+ cells stained with anti-CD3 A-700 m Ab. The number indicated in the dot plot represents the percentage of CD3+ cells that co-stained with *H.pylori*-GFP. The histograms in panel C show the percentage of CD3+ cells that were stained with live (blue line) and fixed (green line) bacterium, compared with unstimulated cells (red line).

To ascertain whether the T cell clustering was due to a direct interaction of the bacteria with purified T lymphocytes, co-cultures were also examined by confocal microscopy, using GFP-transfected *H. pylori*. As shown in Figure 2A, green fluorescent bacteria were tightly surrounding most of the purified T cells. In addition, we assessed by flow cytometry the percentage of purified CD3+ cells that co-localized with GFP fluorescent bacteria. As compared to the control (Figure 2A), about 80% of CD3+cells had fluorescent bacteria bound to them (Figure 2B). Interestingly, this interaction was nearly absent if the bacteria were treated with formaldehyde (Figure 2C). Taken altogether these data strongly suggest that the bacteria directly interacted with T cells and that this interaction required viable *H. pylori*.

### *H. pylori* activation of purified T lymphocytes in short term co-cultures

**Figure 3:**
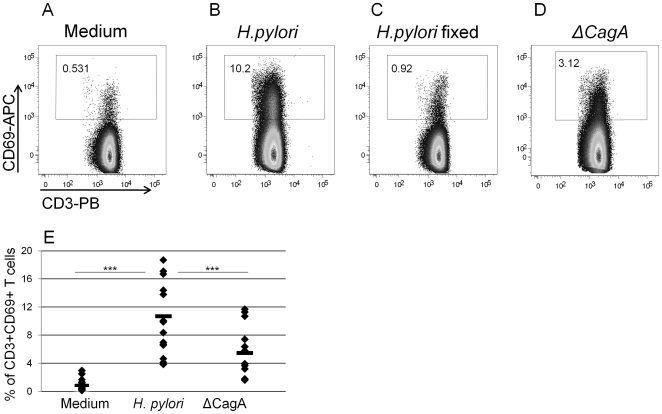
CD3+ T cells activation. Viable *H. pylori* activate purified human peripheral CD3+ T cells *in vitro* in a non-antigen-specific fashion. Purified CD3+ cells were co-cultured with *H. pylori* (MOI 100). After 18 h cells were stained with anti CD3-PB and anti-CD69-APC. Numbers represent the percentage of CD3+CD69+ cells. The difference in CD69 expression in the presence of wild-type *H. pylori* or of the ΔCagA strain (E) was investigated in 15 independent experiments, and was statistically significant (***: P≤0.0001).

To investigate if the observed interaction also modulated the function of T lymphocytes, purified CD3+ cells were co-cultured with viable *H. pylori*, using fixed bacteria as a control, to assess the up-regulation of CD69, known to be an early activation marker antigen of lymphocytes. Figure 3 shows that CD69 was significantly up-regulated by CD3+ cells co-cultured with live bacteria, but not with killed bacteria. These data show that *H. pylori* driven T lymphocytes activation occurred in the absence of APCs, and suggest that this effect was independent of their antigen specificity. Moreover, we also found that the T cell responsiveness was not increased when we used PBMCs from *H. pylori* positive subjects (supplementary materials Figure S1). This suggests that the activation mechanism is not antigen-specific, and it does not depend on previous infections with *H. pylori*.

The ability to induce up-regulation of CD69 on CD3+ cells was also evaluated using a mutant of *H. pylori* G27 unable to synthesize CagA (ΔCagA). It is well known that CagA is translocated into gastric epithelial cells causing changes in cell structure, function and morphology [11]. The CD69 up-regulation was partially reduced when cells were co-cultured with the bacteria lacking CagA, as compared to wild type bacteria (average of 42% of reduction). In conclusion, bacterial viability, rather than integrity is required for CD3+ lymphocytes activation, with CagA being partially involved in this process.

### *H. pylori* induced cytokine production by T cells in the absence of APCs

Subsequently we evaluated whether this activation of CD3+ cells after co-culture with *H. pylori* was accompanied by production of cytokines/chemokines in the supernatants. Indeed, *H. pylori* induced the production of cytokines such as TNFα, IFNγ and chemokines such as MIP1-β, Rantes by CD3+cells. Very low levels of IL-2 were detected; moreover IL-6 was undetactable, indicating that our system was highly purified from APCs. Note that IL-6 was detectable at high levels when unfractionated PBMCs were stimulated with live *H. pylori* (for IL-6: medium  = 33±26 pg/ml versus live *H. pylori*  = 1338±421 pg/ml). This effect was measurable already after 4 hours (Table 1), and increased during overnight stimulation. Production of cytokines and chemokines was confirmed by intracellular staining, after stimulation of purified CD3+ cells for 16 h with viable *H. pylori*. In addition, CD3+ T lymphocytes did not produce cytokines when co-cultured with killed bacteria (data not shown).

**Figure 4:**
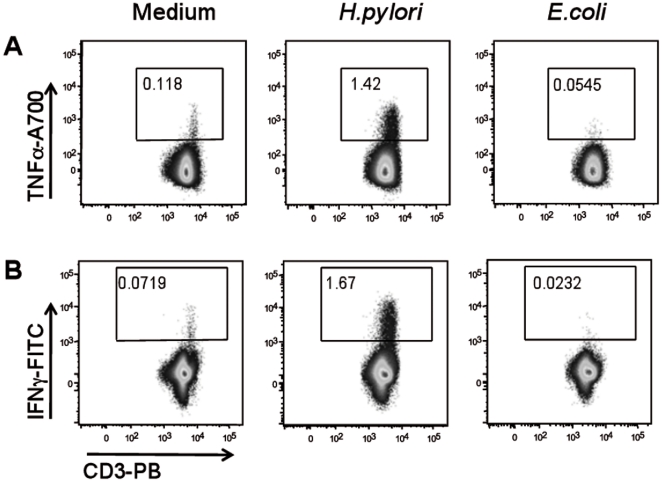
CD3+ T cells and *E.coli* co-culture. *H. pylori* activate CD3+ T cells in a non-antigen-specific fashion after 16 hours of co-culture by inducing cytokines production such as TNFα (A) and IFNγ (B). *E.coli* was not able to exert the same stimulatory effect. Data are representative of three independent experiments with similar results. The numbers in each panel represent the percentage of TNFα and IFNγ-producing CD3+ cells.

To evaluate whether this effect was specific of *H. pylori* or it was shared by other Gram-negative bacteria, some experiments were carried out with *Escherichia coli*. Unlike *H. pylori*, *E.coli* was unable to induce cytokines production by CD3+ T cells (Figure 4), nor CD69 up-regulation (not shown) suggesting that this stimulatory effect was peculiar of *H. pylori*, and not shared with other Gram-negative bacteria.

**Table 1:** CD3+ cells produce cytokines and chemokines after 4 hours of co-culture with *H. pylori*.

	Medium	+ *H. pylori* wt		+ *H. pylori* Δ CagA	
pg/ml	Average (Range)	Average (Range)	*P value*	Average (Range)	*P value*
			*(medium vs H. pylori wt)*		*(H. pylori wt vs ΔcagA)*
**IFN-γ**	12.1 (1.52–27.59)	307.4* (40.74–641.61)	0.049	161.4* (29.74–367.32)	0.030
**TNF-α**	6.0 (5.31–16.26)	476.5* (88.46–882.42)	0.025	344.9* (47.72–717)	0.020
**Rantes**	15.8 (4.7–27.02)	150.9 (42.27–265.89)	0.090	97.5 (16.43–184.7)	0.050
**MIP-1β**	21.7 (4.25–54.16)	968.1* (343–1600)	0.026	392.7* (184.58–901)	0.100
**IL-2**	3.3 (0.33–6.62)	8.1* (6.41–13.42)	0.030	4.2* (2.585–7.71)	0.030
**IL-8**	1.4 (1.84–0.98)	20.1 (14.66–36.12)	0.11	12.5 (6.62–29.65)	0.25
**IL-10**	n.d (<1.3 pg/ml)	n.d (<1.3 pg/ml)	n.a.	n.d (<1.3 pg/ml)	n.a.
**IL-6**	n.d (<1.4 pg/ml)	n.d (<1.4 pg/ml)	n.a.	n.d (<1.4 pg/ml)	n.a.
**IL-17**	2.2 (1.31–6.7)	7.3 (1.31–10.47)	0.22	5.4 (1.36–8.86)	0.26

Purified CD3+ cells from peripheral blood of *H. pylori* negative donors produce a wide range of cytokines and chemokines after 4 h of culture with *H. pylori*. Culture supernatants was collected and analyzed by bioplex assay. Data represent the mean and the range of cytokines and chemokines produced by T cells. Statistical significances of the differences between the cytokines production of CD3+/*H. pylori* co-culture compared to the control group (medium) were assessed using paired t test and reported in the third column. The differences in cytokines production in presence of *H. pylori* wt and ΔCagA were also calculated and compared, as reported in the last column of Table 1. The average was calculated from four independent experiments. Statistical significance was determined by Student's paired T-test at *: P≤0.05. Note: n.d  =  not detectable. n.a = not applicable.

### *H. pylori* induced up-regulation of CD69 and cytokines production by γδ+ T cells in the absence of APCs

**Figure 5:**
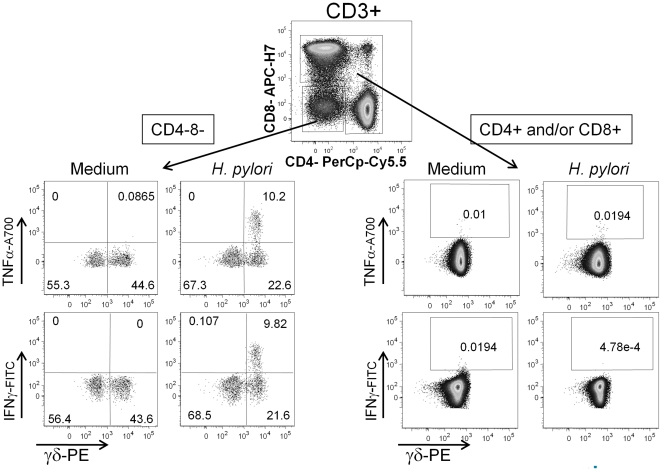
γδ+ T cells activation. γδ+ T cells produce cytokines after co-culture with live *H. pylori* (right panels). Purified CD3+ cells were co-cultured for 18 h with viable bacteria at MOI 100. The first dot plot represents purified CD3+ cells, then dissected into CD4-8- and CD4+ and/or CD8+.

We then asked which CD3+ T cells populations were preferentially activated by *H. pylori*. We observed that the majority of CD3+ producing cytokines were CD3+CD4-CD8- double negative. Therefore we asked whether, among these cells, γδ+ T cells were preferentially activated. We found that 30–60% of CD3+CD4-CD8- T cells were TCR γδ+ and 10% of total γδ+ T cells produced TNFα and IFNγ after co-culture with the bacteria (Figure 5). These γδ+ T cells also produced the chemokines MIP1-β and Rantes and about 90% of them up-regulated the activation marker CD69 (data not shown). It is worth of note that the activation of γδ+ T cells by *H. pylori* clearly was independent on professional APCs, indicating that the mechanism was not antigen specific.

**Figure 6:**
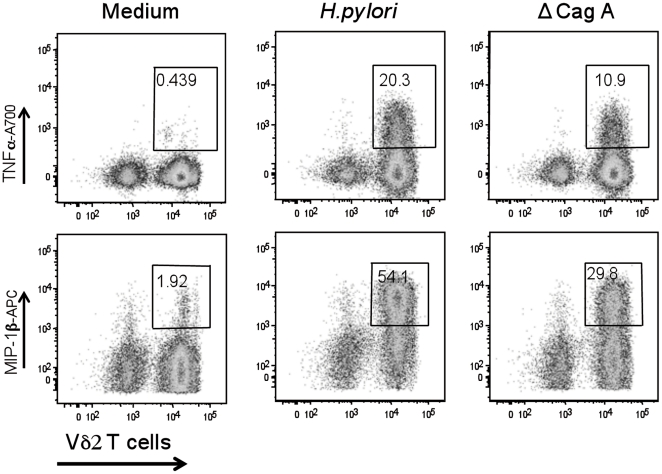
Vγδ2+ cells activation. Among γδ+ cells, Vδ2+ cells are those preferentially activated following co-culture of CD3+ T cells with viable *H. pylori*. Purified CD3+ cells co-cultured with viable *H. pylori* wild type or the ΔCagA mutant at MOI 100 for 18 h. Dot plots are gated on CD3+/γδ+ T cells. Data are representative of three independent experiments with similar results. The numbers in each panel represent the percentage of TNFα and MIP-1β producing γδ+ cells, gated on CD3+ cells.

In order to better characterize the phenotype of γδ+ T cells principally involved in the response to *H. pylori*, we evaluated the contribution of the Vδ2+ T cell subset. This subset of γδ+ T cells has been frequently reported to play a role in immunity against bacterial, parasitic pathogens and tumors [12]. We found that the majority of cytokine secreting CD3+T cells after co-culture with *H. pylori* were Vδ2+ (Figure 6). In addition ΔCagA was still able to induce T lymphocytes activation, although at a level lower than *H. pylori* wild type. Finally, no cytokine production was observed after stimulation of γδ T cells with purified bacterial protein CagA (not shown), indicating that active processes mediated by *H. pylori* were required to achieve the highest activation of T cells and suggesting that CagA had to be actively translocated into the cells to exert its effect.

## Discussion

In this study we show that *H. pylori* is able to stimulate peripheral blood T lymphocytes from *H. pylori* negative donors without the need for APCs. The fact that this effect happens on *H. pylori* negative subjects supports the idea that the stimulation is not antigen specific. This activation requires direct contact between viable bacteria and T cells, suggesting that the binding to the T cell membrane is necessary with a metabolically active bacterium. In particular, we found that live *H. pylori* is a potent activator of peripheral blood γδ+ T cells. Indeed after only 4 hours of co-culture these cells showed significant up-regulation of the activation molecule CD69 and release of a wide range of cytokines (such as TNFα, IFNγ) and chemokines (such as MIP-1β, RANTES). Moreover, the majority of γδ+ T cells producing cytokines expressed the Vδ2 TCR chain. Vδ2+ subset is reported to be involved in the response against a wide range of pathogens [8], [13] although there is still much to understand about the fine antigen specificity of these cells, especially in the context of *H. pylori* recognition.

Our findings that *H. pylori* is able to induce activation of T cells are consistent with a previous report [14] describing murine CD4+ T cell clones that were activated by the bacteria in the absence of APC. However in our study cells activated after contact with bacteria were essentially CD3+CD4-CD8- γδ+ Tcells. This could be explained by the fact that we stimulated freshly isolated human peripheral blood instead of differentiated Th1 and Th2 murine clones. As suggested by the authors, terminally differentiated Th1 and Th2 murine cells could express receptors that allow activation by *H. pylori* [14]. Remain to clarify if also in human the CD4+ T cells after maturation could become more responsive to the *H. pylori* activation, although available evidence with human CD4+ T cell clones does not appear to support the murine data [6].

Since *H. pylori* resides at the apical side of the epithelial layer of the gastric mucosa, one can raise the question on how viable bacteria (and not just bacterial antigens) can interact with lymphoid cells. The interaction between *H. pylori* and T cells can occur after a damage of the epithelium cell layer during infection. The tissue injury can be mediated by the release of many bacterial products, such as the vacuolating toxin VacA, that is able to alter tight junctions, increasing permeability [15] or CagA, which is actively injected into the epithelial cells where it is responsible for the alteration of epithelial cell morphology [16] or via factors intervening at the basolateral side of the epithelium, such as reactive oxygen intermediates induced by bacterial products secreted or released after autolysis [17]. The damage of the gastric barrier may lead to infiltration of *H. pylori* in the sub-mucosa, generating an inflammatory status with infiltration of mononuclear cells [18], [19]. The activation of γδ+ T cells that we observed after *in vitro* stimulation with live *H. pylori* suggests that *in vivo* these cells may recognize and interact directly with the bacteria infiltrating the lamina propria. It has been reported that Vδ2+ cells which are also found in the intestinal epithelium, might contribute to the epithelial homeostasis and might play an important role during the early stage of the immune response against pathogens [12]. Furthermore, some studies have reported an accumulation of γδ+ T cells in the gastric mucosa of *H. pylori* infected subjects that seem to correlate with the severity of gastritis and infiltration of inflammatory cytokines [10].

In the present study we demonstrate, for the first time, that the activation of γδ+ T cells requires viable *H. pylori* and in particular we report that Vδ2+ cell population is affected by this stimulatory effect.

A wide variety of molecules have been described being able to activate Vδ2+ T cells [12], [20], [21]. Among these, there are small compounds (like alkylamines and phosphoantigens) derived from stress-associated surface molecules as well as small bacterial metabolites or microbial compounds produced during infection and cellular stress. The activation of γδ T cells by bacterial metabolites has been demonstrated in several studies using bacterial extract and supernatants containing purified antigens [20], [22]. However, in our experimental conditions we found that *H. pylori* viability is necessary to induce the activation and requires whole bacterium/cell contact. Therefore this leads us to speculate that phosphoantigens or alkylamines are not the major components responsible for this process. Moreover recent reports have shown a functional expression of TLR 2, 3, 5 and 6 in freshly isolated blood Vδ2+ cells [23], [24]. This may suggest that PAMPs, expressed on Gram-negative bacteria surface, could be the putative molecules that activate γδ+ T cells in our *in vitro* system. However, the fact that live *E.coli* or killed *H. pylori* are unable to exert this stimulatory effect tends to rule out an involvement of TLRs agonists in this activation process. Nevertheless, these observations do not exclude the possibility of a partial involvement of TLR agonists as co-receptors. According to that recent studies reported that TCR cross-linking is required for TLR-mediated costimulatory effects on human γδ T cells activation and expansion [25], [26].

Our data support the notion that other *H. pylori* specific components must intervene in the γδ+ T cells activation, which are produced by metabolically active bacteria. CagA represents an ideal candidate, since it is actively inoculated by the bacterium into the epithelial cells via its type IV secretion system [16], [27], [28]. We have shown that the activation of γδ+ T cells by live bacteria is reduced when cells are co-cultured with bacteria lacking CagA. In the present study the activation of γδ+ T cells require live CagA positive bacteria but it was not observed after stimulation with the purified CagA protein alone, suggesting the need for metabolically active bacteria able to mediate translocation of CagA into the lymphocytes. In agreement with the idea that the protein CagA may be internalized by the lymphocytes, as it happens with epithelial cells, we observed by flow cytometry a change in the cellular complexity (Side Scatter) after bacterium-T cell interaction that was nearly absent when T cells were cultured with the strain lacking CagA. The importance of bacterium viability is supported by the fact that treatments leading to kill *H. pylori*, such as formaldehyde fixation, neutralize its stimulatory abilities. On the contrary, the stimulatory activity is not altered after irradiation that makes the bacterium unable to reproduce itself while retaining its vital function. CagA, however, cannot be the only responsible for γδ+ T cells activation, since ΔCagA strains retain their stimulatory ability. Several other factors, such as *H. pylori* type IV organelle, which are not well characterized, could contribute to the *H. pylori* stimulatory ability [29], [30]. Thus, it is likely that contact-dependent secretion of one or more factors transported by the type IV apparatus could actively promote γδ+ T cells activation. Further experiments will be necessary to better clarify all the molecular mechanisms that lie behind *H. pylori*/γδ T cells interaction. Note that experiments of stimulation with VacA-knockout *H. pylori* mutant strain showed that the stimulatory ability of the bacteria was retained even in the absence of VacA (supplementary materials Figure S2).

Our results on the *in vitro H. pylori* activation of γδ+ T cells after short time interaction leads us to speculate that the bacteria could take advantage form this activation, by creating an environment that prevents the complete clearance of the pathogen. In fact, despite the strong immunological response, the pathogen is rarely eliminated and, in the absence of treatment, infection can persists for life. Previous studies have reported that chronic phase of inflammation is also characterized by the concomitant presence of regulatory T cells at the infection site can contribute to *H. pylori* persistence by suppressing antibacterial responses [31]. According to this, we observed that the direct contact of *H. pylori* with T lymphocytes activates T cells and in particular γδ+ T cells to produce CCR5 agonists (such as MIP1-β) that could participate in the recruitment of Tregs at the site of infection, where they may start to exert their suppressive functions. Moreover it has been hypothesized that the Tregs cell homing in sites of infection may be driven by the chemokine receptor CCR5 that is described to be preferentially expressed by Tregs compared with normal CD4+ T cells [32]. In this frame we could speculate that the bacterium activates γδ+ T cells to produce chemokines that may positively control the recruitment of regulatory T cells to the sites of gastric lesions.

Overall our findings showed that *H. pylori* can actively modulate the function of CD3+cells and particularly γδ+ T cells. This activation may turn out to play a role in the maintenance of the local inflammation and eventually to the gastric and duodenal diseases.

## Materials and Methods

### Cell isolation and culture

Buffy coats were obtained from blood of donors serologically negative for *H. pylori*. PBMC were separated by Ficoll density centrifugation (Ge Healthcare, Little Chalfont, United Kingdom). The PBMC layer was recovered, washed and then resuspended in RPMI 1640 complete medium, (RPMI 1640 supplemented with L-glutamine and 25 mM HEPES, containing 5% Human Serum (Celbio, Milan, Italy) or 2% of FBS (HyClone South, Logan, UT).

For the co-culture experiments with *H. pylori*, PBMCs were seeded in 96-well flat-bottom plates at a density of 2×10^6^ cells/well. In all tests performed, T cells viability was assessed over time using flow cytometric analysis and was comparable to that of unstimulated cells.

CD3+ T cells were isolated from PBMCs by magnetic cell separation using the Dynabeads Cell Isolation kits (Invitrogen, San Diego, CA). The purity of cell preparations was confirmed by flow cytometry and was found to be greater than 98%. Some experiments were performed with sorted CD3+, CD3+CD4+, CD3+CD8+, CD3+CD4-CD8- cells using FACSAria cell sorter (BD, Becton, Dickinson Franklin Lakes, NJ). The yield of purification, confirmed by FACS, was greater than 99%. In addition, in some experiments we also used CD14 marker (BD, Becton, Dickinson Franklin Lakes, NJ) to ascertain that our cell preparation did not contain CD14+ cells following magnetic separation and/or cell sorting.

### Culture and preparation of *H. pylori*

*H. pylori* strain G27 and *H. pylori* G27 lacking the CagA gene (ΔCagA) and the VacA gene (ΔVacA) have been extensively previously described [33], [34], [35], [36].

Bacteria were cultured microaerobically, using Campygen gas generating system (Oxoid, Cambridge, UK) for 12 hours at 37°C, on solid media consisting of Tryptic (trypticase) Soy Agar (TSA) plates containing 5% FBS and *H. pylori* selective Agar (DENT), supplemented with 200 µg/ml of kanamycin for the growth of kanamycin-resistant mutants. Bacteria harvested from the plates, were used to inoculate liquid cultures starting from an A_535_ of 0.2 in 15 ml of BHI supplemented with 5%FBS and DENT (+/− Kanamycin) and grown at 37°C in microaerophilic conditions with vigorous shaking (180 rpm) to an A_535_ of 0.5. Bacteria were finally resuspended with BHI and 10 µl of bacteria suspension were added to T cells suspension in order to obtain a multiplicity of infection (MOI) of 100. After 14 h at 37°C under 5% CO_2_ humidified atmosphere, cultures were observed with an optical microscope (Leica, Wetzlar, Germany) with 10x lents. For the formaldehyde fixation, bacteria from liquid culture were incubated in 2% formaldehyde in PBS for 30 min at room temperature and washed four times in PBS. For peptidase digestion, bacteria were incubated for 2.5 hours with proteinase K (Sigma-Aldrich, Taufkirchen, Germany) at final concentration of 2.5 µg/ml. Irradiated *H. pylori* were obtained treating 10^9^ bacteria/ml of PBS at 6000 rad and then washed and resuspended at the working concentration.

### Confocal microscopy

Interaction between cells and *H. pylori* was investigated using Kanamycin, GFP transfected *H. pylori* G27 strain [37], [38], provided by Dr Stefano Censini (Novartis Vaccines and Diagnostics, Siena, Italy).

Interaction between GFP-bacteria and T cells was imaged by Zeiss Observer LSM 710 confocal microscope. DAPI staining was used to determine the number of nuclei and to assess gross T cells morphology. Laser lines at 405 nm, 488 nm were used for excitation of DAPI and GFP respectively.

### Analysis of activation markers and cytokines production by Flow cytometry (FACS)

T cell response was assessed by stimulating purified T cell populations with viable bacteria for 18 hours. Brefeldin (BFA, BD Biosciences, Franklin Lakes, NJ) was added after 2 hours in order to block proteins secretion. PBMC cultures in medium alone were included as negative control.

Cells were stained with the LIVE/DEAD aqua viability marker (Invitrogen), incubated with surface antibodies anti-CD69, fixed and permeabilized with the cytofix/cytoperm kit (BD Biosciences) and incubated with antibodies specific for CD3, CD4, CD8, IL-2, IFNγ, TNFα, Vγδ, Vδ2 TCR (all from BD Pharmingen, San Diego, CA) conjugated with indicated fluorochromes.

Samples were acquired with a FACS LSRII (BD Bioscience) and analyzed using Flowjo analysis software.

### Cytokine secretion

Culture supernatants were harvested after 4 and 16 hours of *H. pylori*/purified T cells co-culture and stored at −20°C until analysis. Cytokine secretion was measured by Bio-Plex assay (Bio-Rad, Hercules, CA), according to manufacturer's instructions using the human 27-plex panel. The following soluble proteins were quantified: IL-1β, IL-1ra, IL-2, IL-4, IL-6, IL-8, IL-10, IL-12p70, IL-13, IL-15, IL-17, eotaxin, basic fibroblast growth factor, G-CSF, GM-CSF, IFN-γ, IP-10, MCP-1 (CCL2), MIP-1α (CCL3), MIP-1β (CCL4), PDGF, RANTES (CCL5), TNF-α, and vascular endothelial growth factor.

### Statistical Analysis

Statistical analysis was done by the paired Student's paired T test with a two-tailed distribution.

## Supporting Information

Figure S1 CD3+ cells from peripheral blood of *H. pylori*-positive (n = 3) donors produce cytokines and chemokines comparable to *H. pylori*-negative subjects (n = 4) (A). Culture supernatants was collected after 4 h of co-culture with *H. pylori* and analyzed by bioplex assay. Data represent the means and the range of cytokines and chemokines produced by T cells. No increase in cytokine and chemokine production was observed with PBMCs from *H. pylori*-positive subjects compared to the *H. pylori* negative. Note: n.d  =  not detectable. B. The percentage of CD69 up-regulation induced by viable *H. pylori* on CD3+ T cells is comparable in *H. pylori*-positive and *H. pylori*-negative subjects. Purified CD3+ cells were co-cultured with *H. pylori* (MOI 100). After 18 h cells were stained with anti CD3-PB and anti-CD69-APC. Numbers represent the percentage of CD3+CD69+ cells. The average was calculated from three independent experiments.
(TIFF)

Figure S2 *H. pylori* ΔVacA activate CD3+ T cells in a non-antigen-specific fashion after 16 hours of co-culture by inducing IFN-γ production. No differences have been found between G27 wild type and *H. pylori* VacA knockout mutant, suggesting that VacA is not involved in this activation mechanism. On the contrary, in the presence of the mutant ΔCagA a reduction of IFN-γ production was observed. Data are representative of two independent experiments with similar results. The numbers in each panel represent the percentage of IFN-γ-producing CD3+ cells.
(TIFF)

## References

[1] Pinto-Santini D, Salama NR (2005). The biology of *Helicobacter pylori* infection, a major risk factor for gastric adenocarcinoma.. Cancer Epidemiol Biomarkers Prev.

[2] Polk DB, Peek RM (2010). *Helicobacter pylori*: gastric cancer and beyond.. Nat Rev Cancer.

[3] Peek RM, Miller GG, Tham KT, Perez-Perez GI, Zhao X (1995). Heightened inflammatory response and cytokine expression in vivo to cagA+ *Helicobacter pylori* strains.. Lab Invest.

[4] Ching CK, Wong BC, Kwok E, Ong L, Covacci A (1996). Prevalence of CagA-bearing *Helicobacter pylori* strains detected by the anti-CagA assay in patients with peptic ulcer disease and in controls.. Am J Gastroenterol.

[5] Blaser MJ, Perez-Perez GI, Kleanthous H, Cover TL, Peek RM (1995). Infection with *Helicobacter pylori* strains possessing cagA is associated with an increased risk of developing adenocarcinoma of the stomach.. Cancer Res.

[6] D'Elios MM, Manghetti M, De Carli M, Costa F, Baldari CT (1997). T helper 1 effector cells specific for *Helicobacter pylori* in the gastric antrum of patients with peptic ulcer disease.. J Immunol.

[7] Newton DJ, Andrew EM, Dalton JE, Mears R, Carding SR (2006). Identification of novel gammadelta T-cell subsets following bacterial infection in the absence of Vgamma1+ T cells: homeostatic control of gammadelta T-cell responses to pathogen infection by Vgamma1+ T cells.. Infect Immun.

[8] Chien YH, Jores R, Crowley MP (1996). Recognition by gamma/delta T cells.. Annu Rev Immunol.

[9] Carding SR, Egan PJ (2002). gamma delta T cells: Functional plasticity and heterogeneity.. Nat Rev Immunol.

[10] Futagami S, Hiratsuka T, Suzuki K, Kusunoki M, Wada K (2006). gammadelta T cells increase with gastric mucosal interleukin (IL)-7, IL-1beta, and *Helicobacter pylori* urease specific immunoglobulin levels via CCR2 upregulation in *Helicobacter pylori* gastritis.. J Gastroenterol Hepatol.

[11] Amieva MR, Vogelmann R, Covacci A, Tompkins LS, Nelson WJ (2003). Disruption of the epithelial apical-junctional complex by *Helicobacter pylori* CagA.. Science.

[12] Bonneville M, Scotet E (2006). Human V gamma 9V delta 2 T cells: promising new leads for immunotherapy of infections and tumors.. Curr Opin in Imm.

[13] Tanaka Y, Morita CT, Nieves E, Brenner MB, Bloom BR (1995). Natural and synthetic non-peptide antigens recognized by human gamma delta T cells.. Nature.

[14] Rosenplanter C, Sommer F, Kleemann P, Belkovets A, Schmidt A (2007). *Helicobacter pylori* polyclonally activates murine CD4(+) T cells in the absence of antigen-presenting cells.. Eur J Immunol.

[15] Papini E, Satin B, Norais N, de Bernard M, Telford JL (1998). Selective increase of the permeability of polarized epithelial cell monolayers by *Helicobacter pylori* vacuolating toxin.. J Clin Invest.

[16] Stein M, Bagnoli F, Halenbeck R, Rappuoli R, Fantl WJ (2002). c-Src/Lyn kinases activate *Helicobacter pylori* CagA through tyrosine phosphorylation of the EPIYA motifs.. Molec Microbiol.

[17] Bagchi D, Bhattacharya G, Stohs SJ (1996). Production of reactive oxygen species by gastric cells in association with *Helicobacter pylori*.. Free Radic Res.

[18] Montecucco C, Rappuoli R (2001). Living dangerously: How *Helicobacter pylori* survives in the human stomach.. Nat Rev Mol Cell Biol.

[19] Dundon WG, de Bernard M, Montecucco C (2001). Virulence factors of *Helicobacter pylori*.. Int J Med Microbiol.

[20] Bukowski JF, Morita CT, Brenner MB (1999). Human gamma delta T cells recognize alkylamines derived from tea beverage, edible plants, and microbes: Implications for innate immunity.. Arthritis Rheum.

[21] Morita CT, Mariuzza RA, Brenner MB (2000). Antigen recognition by human gamma delta T cells: pattern recognition by the adaptive immune system.. Springer Sem Immunopathol.

[22] Feurle J, Espinosa E, Eckstein S, Pont F, Kunzmann V (2002). Escherichia coli produces phosphoantigens activating human gamma delta T cells.. J Biol Chem.

[23] Pietschmann K, Beetz S, Welte S, Martens I, Gruen J (2009). Toll-Like Receptor Expression and Function in Subsets of Human gamma delta T Lymphocytes.. Scand J Immunol.

[24] Beetz S, Marischen L, Kabelitz D, Wesch D (2007). Human gamma delta T cells - Candidates for the development of immunotherapeutic strategies.. Immunol Res.

[25] Shrestha N, Ida JA, Lubinski AS, Pallin M, Kaplan G (2005). Regulation of acquired immunity by gamma delta T-cell/dendritic-cell interactions.. Ann N Y Acad Sci.

[26] Wesch D, Beetz S, Oberg HH, Marget M, Krengel K (2006). Direct costimulatory effect of TLR3 ligand poly(I: C) on human gamma delta T lymphocytes.. J Immunol.

[27] Stein M, Rappuoli R, Covacci A (2000). Tyrosine phosphorylation of the *Helicobacter pylori* CagA antigen after cag-driven host cell translocation.. Proc Natl Acad Sci U S A.

[28] Ren SM, Higashi H, Lu HS, Azuma T, Hatakeyama M (2006). Structural basis and functional consequence of *Helicobacter pylori* CagA multimerization in cells.. J Biol Chem.

[29] Rohde M, Puls J, Buhrdorf R, Fischer W, Haas R (2003). A novel sheathed surface organelle of the *Helicobacter pylori* cag type IV secretion system.. Mol Microbiol.

[30] Aihara M, Tsuchimoto D, Takizawa H, Azuma A, Wakebe H (1997). Mechanisms involved in *Helicobacter pylori*-induced interleukin-8 production by a gastric cancer cell line, MKN45.. Infect Immun.

[31] Lundgren A, Suri-Payer E, Enarsson K, Svennerholm AM, Lundin BS (2003). *Helicobacter pylori*-specific CD4+ CD25 high regulatory T cells suppress memory T-cell responses to *H. pylori* in infected individuals.. Infect Immun.

[32] Yurchenko E, Tritt M, Hay V, Shevach EM, Belkaid Y (2006). CCR5-dependent homing of naturally occurring CD4+ regulatory T cells to sites of Leishmania major infection favors pathogen persistence.. J Exp Med.

[33] Censini S, Lange C, Xiang Z, Crabtree JE, Ghiara P (1996). cag, a pathogenicity island of *Helicobacter pylori*, encodes type I-specific and disease-associated virulence factors.. Proc Natl Acad Sci U S A.

[34] Covacci A, Censini S, Bugnoli M, Petracca R, Burroni D (1993). Molecular characterization of the 128-kDa immunodominant antigen of *Helicobacter pylori* associated with cytotoxicity and duodenal ulcer.. Proc Natl Acad Sci U S A.

[35] Baltrus DA, Amieva MR, Covacci A, Lowe TM, Merrell DS (2009). The complete genome sequence of *Helicobacter pylori* strain G27.. J Bacteriol.

[36] Petersen AM, Sørensen K, Blom J, Krogfelt KA (2001). Reduced intracellular survival of *Helicobacter pylori* vacA mutants in comparison with their wild-types indicates the role of VacA in pathogenesis.. FEMS Immunol Med Microbiol.

[37] Bagnoli F, Buti L, Tompkins L, Covacci A, Amieva MR (2005). *Helicobacter pylori* CagA induces a transition from polarized to invasive phenotypes in MDCK cells.. Proc Natl Acad Sci U S A.

[38] Wang Y, Roos KP, Taylor DE (1993). Transformation of *Helicobacter pylori* by chromosomal metronidazole resistance and by a plasmid with a selectable chloramphenicol resistance marker.. J Gen Microbiol.

