# Supplementary figures and images for: Helicobacter pylori Induces Activation of Human Peripheral γδ+ T Lymphocytes
Source: PLoS One. 2011 Apr 29;6(4):e19324. doi: 10.1371/journal.pone.0019324 (PMC3084806; doi:10.1371/journal.pone.0019324)

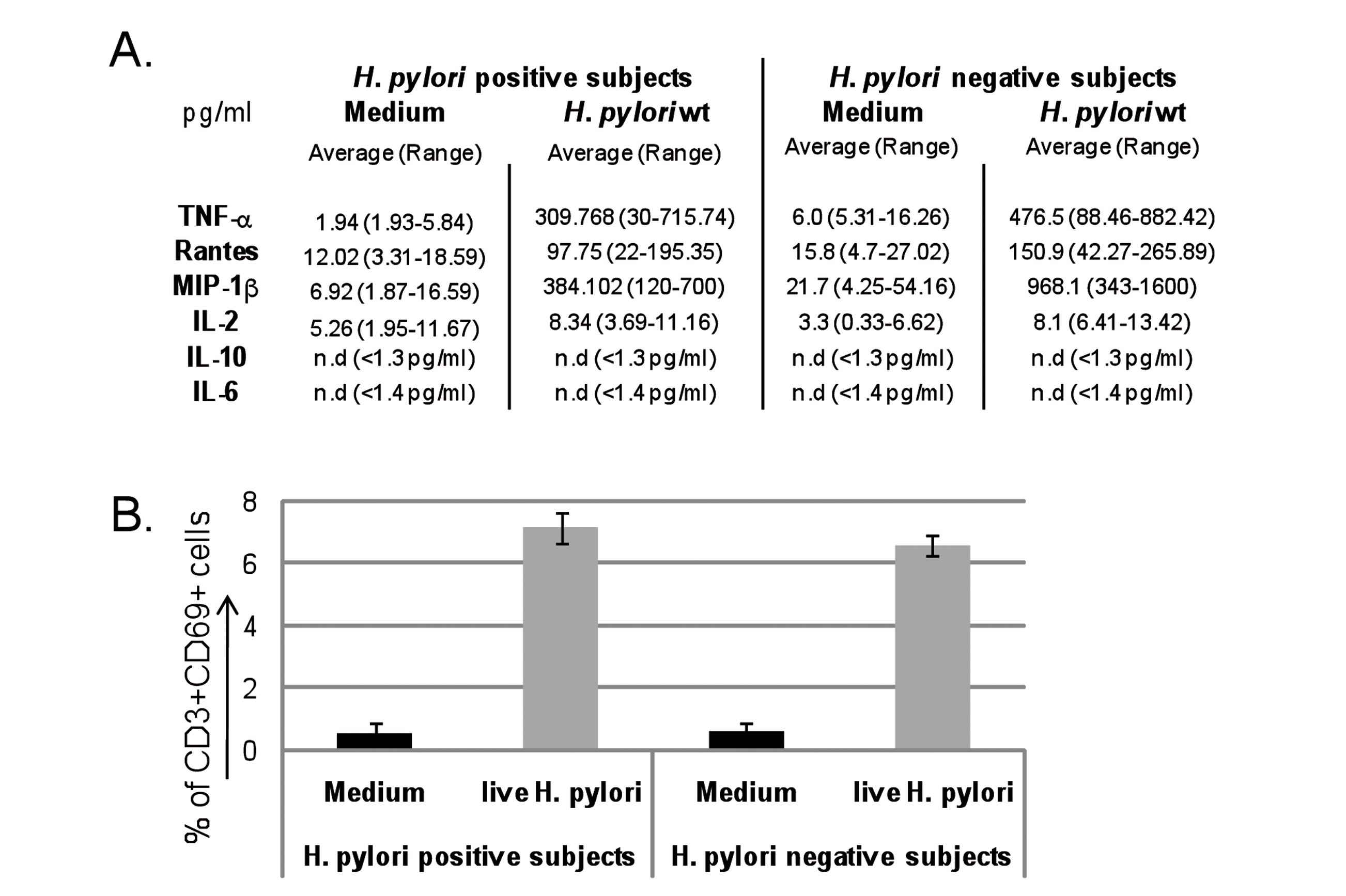

Supplement: Figure S1 — CD3+ cells from peripheral blood of H. pylori-positive (n = 3) donors produce cytokines and chemokines comparable to H. pylori-negative subjects (n = 4) (A). Culture supernatants was collected after 4 h of co-culture with H. pylori and analyzed by bioplex assay. Data represent the means and the range of cytokines and chemokines produced by T cells. No increase in cytokine and chemokine production was observed with PBMCs from H. pylori-positive subjects compared to the H. pylori negative. Note: n.d = not detectable. B. The percentage of CD69 up-regulation induced by viable H. pylori on CD3+ T cells is comparable in H. pylori-positive and H. pylori-negative subjects. Purified CD3+ cells were co-cultured with H. pylori (MOI 100). After 18 h cells were stained with anti CD3-PB and anti-CD69-APC. Numbers represent the percentage of CD3+CD69+ cells. The average was calculated from three independent experiments. (TIFF) [file pone.0019324.s001.tif]

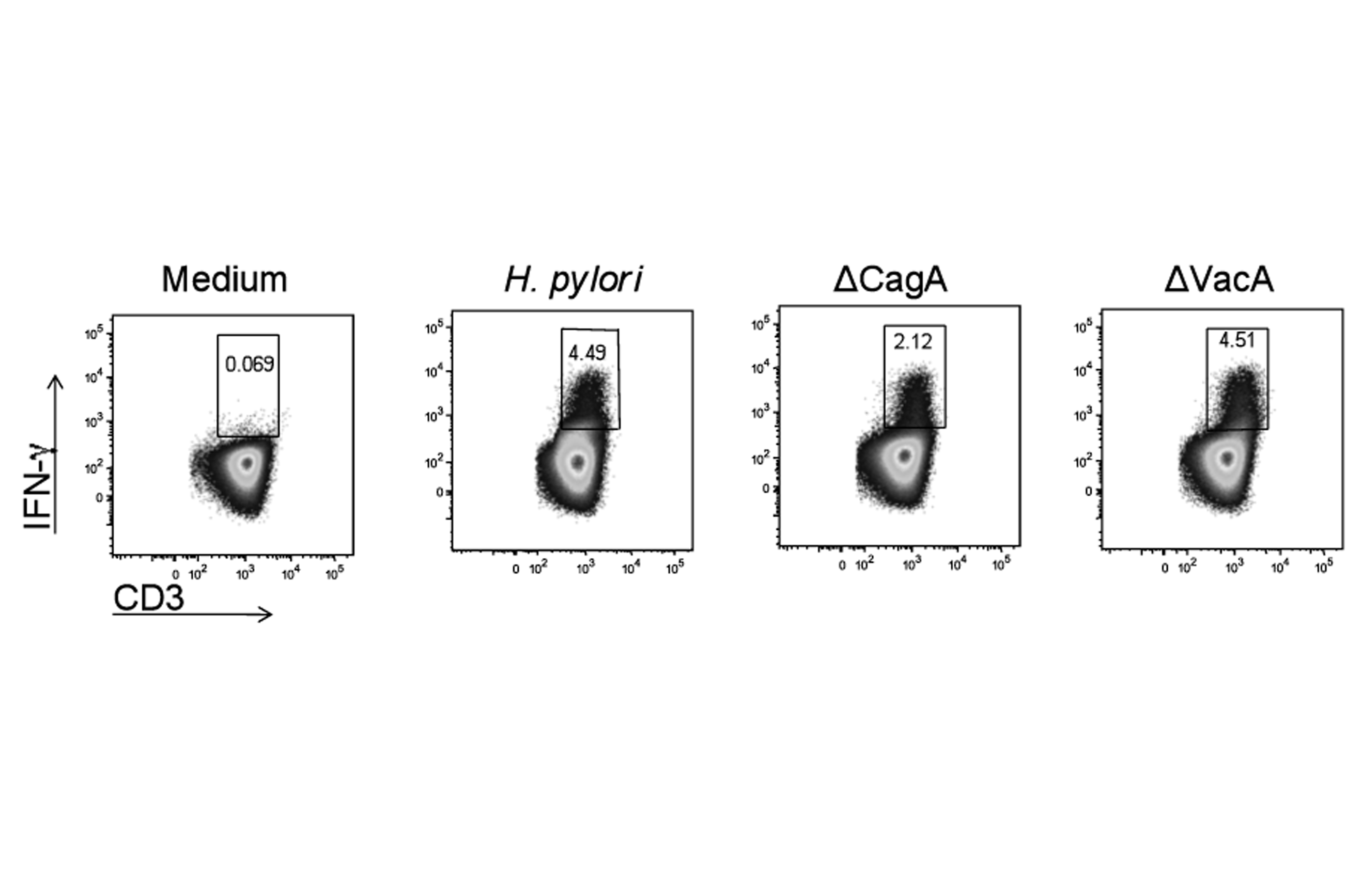

Supplement: Figure S2 — H. pylori ΔVacA activate CD3+ T cells in a non-antigen-specific fashion after 16 hours of co-culture by inducing IFN-γ production. No differences have been found between G27 wild type and H. pylori VacA knockout mutant, suggesting that VacA is not involved in this activation mechanism. On the contrary, in the presence of the mutant ΔCagA a reduction of IFN-γ production was observed. Data are representative of two independent experiments with similar results. The numbers in each panel represent the percentage of IFN-γ-producing CD3+ cells. (TIFF) [file pone.0019324.s002.tif]
